# *“They said we’re all in it together, but we were kind of separated”*: barriers to access, and suggestions for improving access to official information about COVID-19 vaccines for migrants in Australia

**DOI:** 10.1186/s12889-023-15739-z

**Published:** 2023-09-01

**Authors:** Davoud Pourmarzi, Petya Fitzpatrick, Keeley Allen, Aidan Yuen, Stephen Lambert

**Affiliations:** 1 National Centre for Epidemiology and Population Health, Australian National University, Canberra, Australia; 2 National Centre for Immunisation Research and Surveillance, Westmead, Australia; 3 Communicable Diseases Branch, Queensland Health, Brisbane, Australia

**Keywords:** COVID-19, Vaccine, Migrants, Refugees, Trust, Communication

## Abstract

**Background:**

Vaccination is a cornerstone of public health measures to mitigate the burden of COVID-19 infection. Equitable access to information is necessary to ensure all members of society can make an informed decision about COVID-19 vaccines. We sought to investigate barriers that migrants living in Australia faced in accessing official information about COVID-19 vaccines and identify potential solutions.

**Methods:**

This study used a descriptive qualitative study design. Seventeen adults living in Australia and born in the World Health Organization’s Eastern Mediterranean Region participated in a semi-structured interview conducted via telephone. Participants were recruited using advertising through social media platforms. The interviews were conducted between December 2021 and February 2022. All interviews were audio-recorded and transcribed verbatim. Data were analysed using inductive thematic analysis. In this study official information was defined as information provided by Australian Health system.

**Results:**

Barriers to accessing official information about COVID-19 vaccines were related to unmet language needs, methods of dissemination, and mistrust in official sources of information. To overcome barriers, participants suggested improving the quality and timeliness of language support, using diverse modes of dissemination, working with members of migrant communities, providing opportunities for two-way communication, communicating uncertainty, and building a broader foundation of trust.

**Conclusion:**

Information about COVID-19 vaccines during different stages of the vaccination program should be provided in migrants’ languages at the same time that it is available in English using a variety of methods for dissemination. The acceptability of official information can be improved by communicating uncertainty, acknowledging people’s concerns about the safety and effectiveness of COVID-19 vaccines and providing opportunities for two-way communication. People’s trust in official sources of health information can be improved by working with migrant communities and recognising migrants’ contributions to society. The findings of this study may improve managing the response to COVID-19 and other health emergencies in Australia and in other similar societies.

**Supplementary Information:**

The online version contains supplementary material available at 10.1186/s12889-023-15739-z.

## Introduction

Vaccination has been an essential part of public health measures to mitigate the burden of COVID-19 [1]. To ensure health equity, information about vaccines and vaccination programs from official sources must be available to all members of society. This enables people to make informed decisions about COVID-19 vaccines and to protect their health [1]. Accessibility and acceptability of information should be considered in all official communications about COVID-19 vaccines. People who have difficulty accessing official information or who mistrust official sources may rely on information available through sources which are often unregulated and may contain misinformation [2, 3].

The accessibility of information about COVID-19 vaccines is of key importance in multicultural societies such as Australia [4]. Approximately 30% of the Australian population are born overseas. These people are from various cultural, religious, racial and linguistic backgrounds and reflect a range of socioeconomic statuses and English proficiency [5]. Migrants have the right to equitable access to health care so they can protect their health [6, 7]. Access to reliable and accurate health information is a core component of the right to health [6, 7]. Although countries are implementing various initiatives to communicate information about COVID-19 vaccines with different groups of society, there are concerns that certain population subgroups may not be included in all communications appropriately. Ethnic minority groups in high-income countries, including some groups of migrants, are more likely to be excluded from official communications about COVID-19 vaccines [8–10].

Migrant communities in higher-income countries including Australia have disproportionately experienced high rates of COVID-19 incidence, and related hospitalisation and mortality compared with locally born populations [11–15]. Many factors have contributed to this experience including unmet language needs, limited entitlement to health care services, limited access to culturally appropriate services, and systemic discrimination and exclusion from society [11, 16–18]. Providing timely accessible information about COVID-19 vaccines is essential for migrant populations to make informed decisions about vaccines and reduce health inequity [8].

Understanding barriers to accessing official information about COVID-19 vaccines among migrants can help with the design of appropriate communication methods. With this study, we aimed to investigate the barriers experienced by migrants from the World Health Organization’s Eastern Mediterranean Region living in Australia to accessing official information about COVID-19 vaccines and to explore appropriate, acceptable, and community-sourced solutions to overcome barriers.

## Methods

We used descriptive qualitative design to generate in-depth descriptions of migrants’ experiences. People aged 18 years and older, living in Australia, and born in the World Health Organization’s Eastern Mediterranean Region were eligible to participate. Participants were recruited using advertising through social media platforms (primarily Facebook). The study flyer included a link to an online questionnaire where people could read the participant information sheet, and provide consent and their contact details. The questionnaire also collected data on people’s country of birth and if they identified as a health professional or a student to become a health professional. We did not collect other sociodemographic data. People who provided a valid email address or phone number were invited to attend a telephone interview. In selecting participants, we used a maximum variation approach to make sure people born in different countries of the Eastern Mediterranean Region are included and people with health profession backgrounds are not over-represented.

The first author [DP] carried out semi-structured interviews with participants using an interview guide developed based on the aims of the study. The inreview guide included questions related to people access to information about COVID-19 vaccine and the Australian COVID-19 vaccination program and based on the participant’s responses follow-up questions including their suggestions for improving access to official information were asked (Appendix 1). In this study official information was defined as information provided by Australian Health system. DP was born in Eastern Mediterranean Region and is living in Australia. All interviews were conducted in English between December 2021 and February 2022, and each lasted between 20 and 30 minutes. Interviews were audio-recorded and transcribed verbatim. Data were analysed using inductive thematic analysis. Six phases of thematic analysis were applied to analyse the data: familiarisation with data, generating initial codes, searching for themes, reviewing themes, defining and naming themes, and producing the report [19]. A research team member (PF) initiated the coding and discussed the codes and themes with another team member (DP). DP and PF were involved in all aspects of these six phases.

### Ethical consideration

The ethical aspects of this research were approved by the Australian National University Human Research Ethics Committee (Protocol 2021/224). Participants’ information sheet was provided to all participants through an online recruitment questionnaire where participants provided written consent. Before the start of the interview, a verbal consent was also obtained. All participants received a $AUD50 online grocery voucher.

## Results

### Participants

Nine men and eight women, born in 11 different countries, were interviewed. Four participants identified themselves as a health professional or were studying to become a health professional. Four participants during the interview identified themselves as a community leader.

### Barriers

Participants reported barriers were in three main categories including unmet language needs, methods of dissemination, and lack of trust in the government and other authorities. To overcome these barriers participants gave these suggestions: provide timely language support, improve modes of dissemination, communicate uncertainty, and build a broader foundation of trust.

#### Unmet language needs

In the early phase of the vaccine rollout, the lack of translated materials and other language supports posed a significant barrier for some migrants to obtaining accurate and timely information about the COVID-19 vaccines.

*At the beginning of the pandemic, …. the information was just in English, and then it took a while for it to [be] translate it into languages. [W08]*

*If English [is] your second language … it’s hard for you to get right information on right time, … So language barrier is a big thing …, especially our community, … And not many of us speak English. [M02]*

To cope with this problem family and community members took on the responsibility of translating English-language materials into their own language. Sometimes they had difficulty translating medical terms.

*Some of the terminology is not easily translatable in another language. So you have to work your way around some of the terminology, explain it in a different way… a lot of people that I had to talk to, didn’t have that level of education. [W04]*

Furthermore, there was no guarantee that people in need of language support would accept assistance from others. This is particularly important given the uncertainty around COVID-19 vaccines and community and family structure. One participant reported that her father rejected her attempts to assist him and would not agree to be vaccinated until he could read translated official information himself.

*Whatever I say to my dad, he doesn’t really take it very seriously … If I had access to the information in Arabic so that my dad could read, it might have convinced him to get a vaccination earlier. [W02]*

Although the information in other languages was eventually provided through the government translation programme, less common “minority” languages were not supported.

*I see they … have stuff [in] Arabic or Farsi or other languages [but] … information hasn’t been provided in the small community languages. In my community language, I see very rare thing. [M03]*

Furthermore, the poor quality of translations made some translated resources difficult to understand. This undermined people’s ability to trust both this information and the source.

*I noticed that sometimes when the federal government puts out information in Arabic, …. it sounds like gibberish. It doesn’t make sense. … that’s really detrimental. Because then that information … loses all credibility.” [W02]*

#### Mode of dissemination

The methods used to disseminate information about the COVID-19 vaccines created additional access barriers for migrants.

##### Written communication

The government’s reliance on written translated information posed additional barriers for people with low literacy and people from cultures who prefer to communicate orally.

*There are a lot of people even who cannot read their own language. … the community I came from ... most of them, they don’t read. [M02]*

*… but for older generation, … you know they don’t … Some people cannot read or write, some people are more verbal. [W03]*

##### Digital communication

While some participants indicated they preferred to receive information about the vaccines digitally ‘*because it’s easy, and it’s quick’*, the government’s reliance on providing information through digital tools excluded migrants with low digital literacy. Participants pointed out that resources provided on the official websites required people have the capacity to formulate questions that some people, especially the elderly may not have.

*It’s very difficult for elderly people to access it. … My dad had an iPhone for one year, he still doesn’t know how to use it properly. … Just because you have access to something, it doesn’t mean that you’re internet literate, or computer literate or you know how to get the information from the internet that you’re looking for. [W02]*

Even for people who were able to formulate their questions and access the internet, the information overload on official websites and the requirements of high skills in navigating websites affected the accessibility of official information.

*… and even now if you want to go to TGA website, you will get lost. It’s not accessible. It’s very hard to find any information.… because there are many, many text, and many icons on website, and you will get lost. [M05]*

##### One-way communication

One-way didactic messaging from impersonal sources used in many government communications was not deemed acceptable by some who had questions about vaccines and felt powerless to discuss their concerns with health professionals who were not culturally competent.

*You know, government is working a lot,… based you telling the people that vaccine is good, blah, blah. But how about if I have a question, and I don’t have the ability to discuss this with my health care professional, [who] is not from my culture, and I can’t express my feelings and properly discuss my concerns. [M02]*

#### Mistrust in official sources

Participants reported that mistrust in government among migrants affected their acceptance of official sources. In part, this mistrust stemmed from racist attitudes they observed in the Australian government and their experiences of racism, exclusion and alienation from Australian society.

*My mum is … a refugee in the 70s. And the first week I arrived in Australia, [politician’s name] was on TV saying it was a mistake to bring [community’s name] refugees in the 70s to Australia. I can’t believe anything [politician’s name] says and forever after he said that on TV. [W02]*

*We cannot blame the health system, unless we will fix the problem at the top … You cannot treat Muslim communities as … the enemy within all the time and all of the sudden, you would like to have accountability and effectiveness and efficiency when a global epidemic will come. [M07]*

For some people, past experiences of their home country government corruption or war in their home country caused them to mistrust all government officials.

*And it’s probably to do a lot with the background of people. We come from a war torn countries where we couldn’t trust the government. [W08]*

Neglect of migrants’ needs during the early phase of the COVID-19 vaccination program, the experience of being treated differently during the pandemic, along with the perception that governmmnet doesn’t recognise the impact of migrants on the development of the society also led to mistrust in officials.

*“They said, we’re all in it together…. But we were kind of separated from the rest of the community”. [W08]*

*“I think the issue with the government is not recognizing the impact of the migrants [development of society] … in this country… And not you know … rolling out the vaccine for six months, and you’re still not ready address these issues”. [M08]*

### Solutions

#### Provide timely language support

Participants suggested that translated information should be distributed at the same time as English-language resources are, to ensure migrants have adequate time to understand and evaluate it. They also suggested that language support could also be improved in other aspects of migrants’ life.

*The information should not just [be] in terms of the vaccines, health wise, a lot of other aspects of their lives should be provided in their own language, and should be provided in a timely manner. [M08]*

Participants also emphasised that materials should be professionally translated and checked for quality.

*Like actual people, rather than yeah, using Google translation, like actual people should do it. [M09]*

#### Improvemodes of dissemination

##### Use diverse modes of dissemination

To meet the diverse needs of migrant communities, a variety of channels and formats should be used. Participants recommended providing information in audio and video formats, using community-specific media and social media, and using paper-based communication.

*They can’t read, but then those videos that come in through WhatsApp are actually the source of information. … the only thing that’s missing is [the government] needs to work on the WhatsApp. [W03]*

*… like radio channels that are specific to the Arabic language. … they’re a big audience of that. … because they watch TV, they listen to radio, they watch YouTube. [W06]*

*Make some brochures, because I’ve seen here elderly, they are more inclined toward reading and looking at the stuff like newspapers or brochures which are dropped into their houses. [W05]*

##### Engage prominent and trusted members of communities

Participants emphasised the need for the government to collaborate with prominent and trusted members of communities who understand their community’s culture, information needs, and preferred modes for receiving information.

*Work with the community itself, work with the community elders, work with community leaders, work with faith leaders. Because they know … people and what works for them. …., they know what to do for the community, you have that relationship already in place. [M02]*

##### Engage young people

Younger members of communities can be engaged to distribute information as they experience fewer barriers to accessing official information because of their English and digital literacy.

*Focusing on the younger immigrants’ kids. Because I think they have the most access to the web and things like that. Maybe targeting them like ‘did your loved one get the vaccine?” [W06]*

##### Provide opportunities for two-way communications

Given the uncertain and changing nature of information available about the COVID-19 vaccines, and the prevalent concern about risk, participants suggested that educational ‘conversations’ about the vaccines should form part of an overall strategy to overcome access barriers to official information.

*It should be a two-way communication …, in two ways communication is not just you telling me that the vaccine is safe. I have my own concerns that need to be addressed. [M08]*

#### Clearly communicate uncertainty

Participants recommended that when there are changes to official messages about COVID-19 and its vaccines the reasons for these changes should be explained in clear and simple language. This will help people to stay informed and maintain trust in official sources.

*Maybe explanations as to why it’s changing in very basic way just because I feel like a lot of them, because they don’t have that high level of education to sort of get over the lingo and terminology, that sort of, like almost intuitive sense. [M04]*

#### Build a broader foundation of trust

Participants emphasised that the underlying causes of mistrust in migrant communities should be addressed, both during the pandemic and beyond. Suggestions included ongoing consultations with migrant communities and addressing the problem of social exclusion.

*You have to establish the trust way long before pandemic would be happening. … building bridge and being friend with all the communities, …, treat people based on their potential and what they have. [M07]*

## Discussion

Barriers to accessing official information about COVID-19 vaccines among migrants were related to unmet language needs, modes of dissemination, and mistrust in official sources. Providing timely language support, improving the modes of dissemination, communicating uncertainty, and building a broader foundation of trust were suggested by participants to overcome the reported barriers.

Official sources of information should be the main sources that people use to make an informed decision about COVID-19 vaccines. In a survey on the same population, 17% reported that official sources were not their main sources of receiving information about COVID-19 vaccines and more than 80% reported that one of their main source of information was unofficial sources such as family and friends [20]. When official sources are not accessible, people may turn to unofficial sources to find answers to their questions. These sources are more likely to contain misinformation which can contribute to vaccine hesitancy [2, 3].

The Australian health system requires individuals to have adequate health literacy and health system literacy in order to use the available services appropriately to protect their health [21, 22]. Health information is typically provided in written form in English and disseminated digitally. Individuals are responsible for accessing and interpreting information, and taking appropriate actions for their health. This approach excludes people who may not have that level of English language proficiency and digital literacy. Approximately 14% of Australians can only read English at a primary school equivalent level with about 4% at pre-primary school level [23]; 28% are highly excluded or excluded digitally [24] and 21% use languages other than English at home [25].

In communicating public health information, a community engagement approach needs to be used [26, 27]. This is especially important at the time of a health crisis such as COVID-19 when providing timely information is crucial [28]. Using a community engagement approach, the health information needs of the population should be understood and appropriate modes and formats used to reach all members of society [29]. There is no doubt that in a multicultural society such as Australia, information about COVID-19 vaccines should be available in people’s languages. To improve the acceptability of official sources of information, it is important that professional translation services are in place to provide information in different languages at the same time as the English version [30].

Prominent and trusted members of communities should be engaged to give advice about appropriate modes of providing information. These individuals have knowledge about their communities and established trust networks [26, 27, 31]. In most Australian states and territories there are community-specific organisations that provide social support for their communities, try to improve the connection among community members, and support their integration within Australian society. There are also religion-specific organisations that provide religious and social support for their members. These organisations can be approached to find appropriate methods of providing health information for different communities. Engaging members of communities should not be seen as a one fits all model as migrant communities are diverse; migrants from the same country may have different needs and preferences [26].

Engaging younger members of migrant communities can help with the distribution of information about COVID-19 vaccines. Young members of migrant communities usually have better access to health information as they experience fewer language barriers and have higher digital literacy [32]. However, as reported in this study, the structure of the family in different migrant communities should be considered. Previous studies showed the importance of engaging young migrants to prevent the spread of misinformation in a multicultural society [32].

Health inequity can be seen in COVID-19 cases due to a range of reasons, including being disproportionally affected by misinformation. In the context of the newness of COVID-19 and its vaccines, clear communication about uncertainty is necessary. This will help people understand what is known and unknown about COVID-19 vaccines in different stages of the vaccination program, and what actions are undertaken to have more and better quality information [33]. This communication should be two-way, providing opportunities for people to ask questions and express their concerns. If uncertainty is not appropriately communicated this can affect individuals’ trust in official information [34].

There is no doubt that people do not access information if they don’t trust the source. As reported in this study migrants’ mistrust of official prevented them from accessing official information. There are many factors that contribute to the development of mistrust in officials, and migrants in Australia may have a variety of reasons for their mistrust. Generally, non-English speaking migrants have higher trust in the Australian government compared to other Australians [35]. However, negative experiences with the government in their home countries and the Australian government, and experience of racism, exclusion and alienation from Australian society were reported as factors that contributed to mistrust [36]. Experiences of racism, exclusion, and discrimination are commonly reported by migrants living in Australia. A recent survey in 2021 has shown that more than 40% of Australians have negative views about migrants from some Eastern Mediterranean Region countries. It also reported that 34% of people born in non-English speaking countries experienced discrimination because of their skin colour, ethnic origin or religion [35]. It is important that migrants’ experiences with the government, the health system and the general public are considered in health communications. Although engageming trusted community members can help to provide information for migrants who may not trust officials, it is important to build the foundations of trust with migrants, forming connections with migrant communities and recognising migrants’ potential and existing contribution to the development of the society.

### Strengths and limitations

Diversity among participants of this study enabled us to explore the experiences of a diverse group of individuals and communities. Participants of this study included individuals born in 11 different countries and health professionals and members of communities who actively supported their communities during COVID-19 pandemic. However, the requirement to speak English during the interview might have limited people’s willingness or ability to participate or share their experiences. Including participants who identified themselves as a community leader and could speak on behalf of their community might have reduced the impact of this limitation on collected data. Using qualitative methods, we were able to explore people’s experiences and viewpoints. The qualitative nature of the study may limit the applicability of findings in other contexts.

## Conclusion

Official organisations should provide information about COVID-19 vaccines at different stages of the vaccination programme rollout. Information should be available in people’s languages at the same time it is available in English, and be disseminated using methods, channels and formats that improve equity of access. The acceptability of official information can be improved by communicating uncertainty, acknowledging people’s concerns about the safety and effectiveness of COVID-19 vaccines, and providing opportunities for two-way communication. Other steps that will improve the acceptability of the official information include being aware of migrants’ experience with official authorities in their home countries and in Australia, engageing of trusted members of communities, working with the communities, and building a foundation of trust. These findings can help with effective engagement of migrant communities in Australia in all stages of COVID-19 control. The findings of this study may also be applied to other emerging health issues and crises in Australia and other similar societies.

### Electronic supplementary material

Below is the link to the electronic supplementary material.

Supplementary Material 1

## Data Availability

The datasets generated and/or analysed during the current study are not publicly available due to privacy issues but are available from the corresponding author on reasonable request.

## Declarations

All methods were carried out in accordance with relevant guidelines and regulations or Declaration of Helsinki.

## References

[1] World Health Organization. Statement for healthcare professionals: How COVID-19 vaccines are regulated for safety and effectiveness (Revised. March 2022) Geneva: World Health Organization, 2022 [Available from: https://www.who.int/news/item/17-05-2022-statement-for-healthcare-professionals-how-covid-19-vaccines-are-regulated-for-safety-and-effectiveness.

[2] Puri N, Coomes EA, Haghbayan H, Gunaratne K (2020). Social media and vaccine hesitancy: new updates for the era of COVID-19 and globalized infectious diseases. Hum Vaccines Immunotherapeutics.

[3] Pourmarzi D, Fitzpatrick P, Lambert S. “And a huge factor is… the people around them”: Sources of information about COVID-19 vaccines among migrants in Australia. Pre-print. 2022. 10.1177/17579759251334391 PMC13161489 40346811

[4] Wild A, Kunstler B, Goodwin D, Onyala S, Zhang L, Kufi M (2021). Communicating COVID-19 health information to culturally and linguistically diverse communities: insights from a participatory research collaboration. Public Health Research & Practice.

[5] Australian Bureau of Statistics. Migration, Australia Canberra2022 Available from: https://www.abs.gov.au/statistics/people/population/migration-australia/2019-20.

[6] International Organization for Migration. Migration Health in the Sustainable Development Goals: Leave No One Behind’ in an increasingly mobile society Geneva: International Organization for Migration. 2020 [Available from: https://www.iom.int/resources/migration-health-sustainable-development-goals-1.

[7] World Health Organinsation. Human rights and health Geneva: World Health Organinsation. 2017 Available from: https://www.who.int/news-room/fact-sheets/detail/human-rights-and-health.

[8] World Health Organization. Strengthening COVID-19 vaccine demand and uptake in refugees and migrants Geneva: World Health Organization. 2022 Available from: https://www.who.int/publications/i/item/WHO-2019-nCoV-immunization-demand_planning-refugees_and_migrants-2022.1.

[9] World Health Organisation. COVID-19 immunization in refugees and migrants: principles and key considerations Geneva: World Health Organisation. 2021 Available from: https://apps.who.int/iris/bitstream/handle/10665/344793/WHO-2019-nCoV-immunization-refugees-and-migrants-2021.1-eng.pdf.

[10] Immordino P, Graci D, Casuccio A, Restivo V, Mazzucco W (2022). COVID-19 vaccination in Migrants and Refugees: Lessons Learnt and Good Practices. Vaccines.

[11] Greenaway C, Hargreaves S, Barkati S, Coyle CM, Gobbi F, Veizis A (2020). COVID-19: exposing and addressing health disparities among ethnic minorities and migrants. J Travel Med.

[12] Hayward SE, Deal A, Cheng C, Crawshaw A, Orcutt M, Vandrevala TF (2021). Clinical outcomes and risk factors for COVID-19 among migrant populations in high-income countries: a systematic review. J migration health.

[13] Jaljaa A, Caminada S, Tosti ME, D’Angelo F, Angelozzi A, Isonne C (2022). Risk of SARS-CoV-2 infection in migrants and ethnic minorities compared with the general population in the European WHO region during the first year of the pandemic: a systematic review. BMC Public Health.

[14] Ross J, Diaz CM, Starrels JL. The Disproportionate Burden of COVID-19 for Immigrants in the Bronx, New York. JAMA Internal Medicine. 2020;180(8):1043-4. 10.1001/jamainternmed.2020.2131 32383754

[15] Wright L, Steptoe A, Fancourt D (2020). Are we all in this together? Longitudinal assessment of cumulative adversities by socioeconomic position in the first 3 weeks of lockdown in the UK. J Epidemiol Commun Health.

[16] Crawshaw AF, Farah Y, Deal A, Rustage K, Hayward SE, Carter J et al. Defining the determinants of vaccine uptake and undervaccination in migrant populations in Europe to improve routine and COVID-19 vaccine uptake: a systematic review. Lancet Infect Dis. 2022;22(9). 10.1016/S1473-3099(22)00066-4 PMC9007555 35429463

[17] Knights F, Carter J, Deal A, Crawshaw AF, Hayward SE, Jones L (2021). Impact of COVID-19 on migrants’ access to primary care and implications for vaccine roll-out: a national qualitative study. Br J Gen Pract.

[18] Lin S. COVID-19 Pandemic and Im/migrants’ Elevated Health Concerns in Canada: Vaccine Hesitancy, Anticipated Stigma, and Risk Perception of Accessing Care. J Immigr Minor Health. 2022;24(4). 10.1007/s10903-022-01337-5 PMC8874751 35212825

[19] Braun V, Clarke V (2006). Using thematic analysis in psychology. Qualitative Res Psychol.

[20] Allen KLS, Yuen A, Pourmarzi D. Factors associated with COVID-19 booster vaccine willingness among migrants from the Eastern Mediterranean living in Australia: a cross-sectional study. BMC Public Health. 2022;22(1). 10.1186/s12889-022-14608-5 PMC9706861 36443806

[21] Australian Institute of Health and Welfare. Australia’s health 2016. Canberra: AIHW. 2016 Available from: https://www.aihw.gov.au/reports/australias-health/australias-health-2016/contents/summary.

[22] Australian Commission on Safety and Quality in Health Care. Health literacy: Taking action to improve safety and quality Sydney: Australian Commission on Safety and Quality in Health Care. 2014 Available from: https://www.safetyandquality.gov.au/sites/default/files/migrated/Health-Literacy-Taking-action-to-improve-safety-and-quality.pdf.

[23] Australian Government. Literacy and access 2022. Available from: https://www.stylemanual.gov.au/accessible-and-inclusive-content/literacy-and-access.

[24] Thomas J, Barraket J, Parkinson S, Wilson C, Holcombe-James I, Kenned J, et al. Australian digital inclusion Index Melbourne. RMIT, Swinburne University of Technology, and Telstra; 2021.

[25] Australian Bureau of Statistics. Census reveals a fast changing, culturally diverse nation 2016 Available from: https://www.abs.gov.au/ausstats/abs@.nsf/lookup/Media%20Release3.

[26] Migration Council Australia, Supporting. COVID-19 Vaccination Program rollout to migrant and refugee communities in Australia Canberra: Migration Council Australia. 2022 Available from: https://socialpolicy.org.au/wp-content/uploads/2022/02/Policy-brief-Supporting-COVID-19-Vaccination-Program-rollout.pdf.

[27] Wild A, Kunstler B, Goodwin D, Onyala S, Zhang L, Kufi M (2021). Communicating COVID-19 health information to culturally and linguistically diverse communities: insights from a participatory research collaboration. Public Health Research & Practice.

[28] Gilmore BNR, Tchetchia A, de Claro V, Mago E, Diallo AA et al. Community engagement for COVID-19 prevention and control: a rapid evidence synthesis. BMJ Global Health. 2020(10):e003188. 10.1136/bmjgh-2020-003188 PMC7554411 33051285

[29] World Health Organization. Conducting community engagement for COVID-19 vaccines Geneva: World Health Organization. 2021 Available from: https://apps.who.int/iris/handle/10665/339451.

[30] Vieira LNOHM, O’Sullivan C (2021). Understanding the societal impacts of machine translation: a critical review of the literature on medical and legal use cases. Inform Communication Soc.

[31] Kamal A, Hodson A, Pearce JM (2021). A rapid systematic review of factors influencing COVID-19 vaccination uptake in minority ethnic groups in the UK. Vaccines.

[32] Couch J, Liddy N, McDougall J (2021). Our voices aren’t in lockdown’—Refugee young people, challenges, and innovation during COVID-19. J Appl Youth Stud.

[33] Capurro G, Jardine CG, Tustin J, Driedger M (2021). Communicating scientific uncertainty in a rapidly evolving situation: a framing analysis of canadian coverage in early days of COVID-19. BMC Public Health.

[34] Hyland-Wood B, Gardner J, Leask J, Ecker UK (2021). Toward effective government communication strategies in the era of COVID-19. Humanit Social Sci Commun.

[35] Markus A (2021). Mapping Social Cohesion.

[36] Ziersch A, Due C, Walsh M, Discrimination (2020). A health hazard for people from refugee and asylum-seeking backgrounds resettled in Australia. BMC Public Health.

