# Supplementary material for: “They said we’re all in it together, but we were kind of separated”: barriers to access, and suggestions for improving access to official information about COVID-19 vaccines for migrants in Australia
Source: BMC Public Health. 2023 Sep 1;23:1690. doi: 10.1186/s12889-023-15739-z (PMC10472572; doi:10.1186/s12889-023-15739-z)
Supplement: Supplementary file 1 — Supplementary Material 1 [file 12889_2023_15739_MOESM1_ESM.docx]

**Interview guide**

Do you think enough information about the COVID-19 vaccines is provided to you or people around you by the Australian Health System?

How about information about the vaccination program?

Do you think enough information about the Australian COVID-19 vaccination program is provided to you or people around you? For example Eligibility criteria, when to get the vaccine or where to get vaccines or you need to have a booster dose, vaccines for kids.

What information do you think should be provided about COVID-19 vaccines?

How do you usually find information about the COVID-19 vaccine?

Did you experience any problems accessing the information about COVID-19 vaccines?

How can the Australian Health System make sure that enough information about COVID-19 vaccines is available for people like you?
